# Polygenic risk for circulating reproductive hormone levels and their influence on hippocampal volume and depression susceptibility

**DOI:** 10.1016/j.psyneuen.2019.04.011

**Published:** 2019-08

**Authors:** Demelza M. Smeeth, Danai Dima, Lisa Jones, Ian Jones, Nick Craddock, Michael J Owen, Marcella Rietschel, Wolfgang Maier, Ania Korszun, John P. Rice, Ole Mors, Martin Preisig, Rudolf Uher, Cathryn M. Lewis, Sandrine Thuret, Timothy R. Powell

**Affiliations:** a Department of Basic and Clinical Neuroscience, Institute of Psychiatry, Psychology and Neuroscience, King’s College London, London, UK; b Department of Psychology, School of Arts and Social Sciences, City, University of London, London, UK; c Department of Neuroimaging, Institute of Psychiatry, Psychology and Neuroscience, King's College London, London, UK; d Institute of Health & Society, University of Worcester, Worcester, UK; e MRC Centre for Neuropsychiatric Genetics and Genomics, Neuroscience and Mental Health Research Institute, Cardiff University, Cardiff, UK; f Department of Psychiatry, University of Bonn, Bonn, Germany; g Division of Genetic Epidemiology in Psychiatry, Central Institute of Mental Health, Mannheim, Germany; h Barts and The London Medical School, Queen Mary University of London, London, UK; i Department of Psychiatry, Washington University, St. Louis, Missouri, USA; j Psychosis Research Unit, Aarhus University Hospital, Risskov, Denmark; k University Hospital Center and University of Lausanne, Lausanne, Switzerland; l Department of Psychiatry, Dalhousie University, Halifax, Nova Scotia, Canada; m Social, Genetic and Developmental Psychiatry Centre, Institute of Psychiatry, Psychology and Neuroscience, King’s College London, London, UK

**Keywords:** Reproductive hormones, Estradiol, Hippocampal volume, Postpartum depression, Major depression, Polygenic risk scores

## Abstract

Altered reproductive hormone levels have been associated with the pathophysiology of depressive disorders and this risk may be imparted by their modulatory effect upon hippocampal structure and function. Currently it is unclear whether altered levels of reproductive hormones are causally associated with hippocampal volume reductions and the risk of depressive disorders. Here, we utilize genome-wide association study (GWAS) summary statistics from a GWAS focusing on reproductive hormones, consisting of 2913 individuals. Using this data, we generated polygenic risk scores (PRS) for estradiol, progesterone, prolactin and testosterone in the European RADIANT cohort consisting of 176 postpartum depression (PPD) cases (100% female, mean age: 41.6 years old), 2772 major depressive disorder (MDD) cases (68.6% female, mean age: 46.9 years old) and 1588 control participants (62.5% female, mean age: 42.4 years old), for which there was also a neuroimaging subset of 111 individuals (60.4% female, mean age: 50.0 years old). Only the best-fit PRS for estradiol showed a significant negative association with hippocampal volume, as well as many of its individual subfields; including the molecular layer and granule cell layer of the dentate gyrus, subiculum, CA1, CA2/3 and CA4 regions. Interestingly, several of these subfields are implicated in adult hippocampal neurogenesis. When we tested the same estradiol PRS for association with case-control status for PPD or MDD there was no significant relationship observed. Here, we provide evidence that genetic risk for higher plasma estradiol is negatively associated with hippocampal volume, but this does not translate into an increased risk of MDD or PPD. This work suggests that the relationship between reproductive hormones, the hippocampus, and depression is complex, and that there may not be a clear-cut pathway for etiology or risk moderation.

## Highlights

• Hippocampal volume and depression risk have been linked to reproductive hormones.
• We generated polygenic risk scores (PRS) for four reproductive hormones.
• PRS for higher estradiol predicted smaller hippocampal subfield volumes.
• None of the PRSs predicted postpartum or major depression risk.

## 1 Introduction

The hippocampus, like the rest of the brain, changes in volume throughout the human lifespan and is subject to factors such as age (Daugherty et al., 2016), psychiatric health (Harrisberger et al., 2015) and altered circulating endogenous factors, such as hormones (Barth et al., 2016). The hippocampus is not a homogeneous structure and is composed of multiple subfields, each with a different cellular makeup, function and associated circuitry (Knierim, 2015). Cellular mediators of hippocampal volume changes include altered cell death, cell size, and branching of both neurons and astroglia (Czéh and Lucassen, 2007). Additionally, adult hippocampal neurogenesis (AHN) provides a unique region-specific mechanism contributing to variability in the volume and plasticity of the dentate gyrus, specifically (Toda and Gage, 2018).

Research has shown that the endocrine system is partly involved in the regulation of hippocampal volume and function, indicating roles for estrogens (i.e. estradiol), androgens (i.e. testosterone), progestogens (i.e. progesterone) and peptide hormones (i.e. prolactin) (Galea et al., 2013). A variety of cross-sectional, intervention and longitudinal studies have shown that these circulating reproductive hormones often correlate with hippocampal volume (Barth et al., 2016; Bayer et al., 2013; Braden et al., 2017; Panizzon et al., 2018; Seiger et al., 2016). The mammalian hippocampus has the potential to be a direct target of reproductive hormones as it expresses the receptors necessary for signal transduction, namely the estrogen receptors 1 and 2, the androgen receptor, progesterone receptor and prolactin receptor (Cabrera-Reyes et al., 2017; Meffre et al., 2013; Shughrue and Merchenthaler, 2000; Tsai et al., 2015). In the hippocampus, downstream signaling pathways of these receptors have the potential to drive volume changes through their known effects on AHN, dendritic morphology and the survival of neurons (Galea et al., 2013; Mahmoud et al., 2016).

The volume of the hippocampus and its subfields have been linked to a wide range of neurological and psychiatric disorders. Reduced hippocampal volume is observed in anxiety, schizophrenia, Alzheimer’s disease, and the aged brain in general (Arnold et al., 2015; Daugherty et al., 2016; van Erp et al., 2016; Hibar et al., 2016; Schoenfeld et al., 2017; Schuff et al., 2009). A loss of hippocampal volume is a commonly reported feature of depressive disorders (Campbell et al., 2004; Schmaal et al., 2016; Sheline et al., 1996) and is not only associated with the presence of the disease, but volume reductions often correlate with increased disease severity or recurrence (Schmaal et al., 2016; Treadway et al., 2015). It is hypothesized that these volume reductions mediate disturbances in cognitive function (e.g. negative affect, ruminating thoughts) which in turn, increase risk for depressive disorders (MacQueen and Frodl, 2011). In addition, individual hippocampal subfields exhibit independent volumetric changes in a variety of contexts. For example, atrophy of the dentate gyrus and cornu ammonis regions is commonly observed in major depressive disorder (MDD) (Huang et al., 2013) and schizophrenia (Haukvik et al., 2015), but only schizophrenia patients exhibit smaller subiculum regions (Haukvik et al., 2015).

Changes to circulating reproductive hormones have also been linked to the development of depressive disorders in a wide range of human studies (Balzer et al., 2015; Faron-Górecka et al., 2013; Holsen et al., 2011; Schiller et al., 2015; Zarrouf et al., 2009). Interestingly, females have an almost two-fold greater risk of developing MDD relative to men (Bromet et al., 2011; Kessler et al., 1993), which is thought to be in part due to hormonal differences and the prevalence of reproductive-related depressive disorders such as postpartum depression (PPD) (Kuehner, 2017). Due to the aforementioned link between reproductive hormones and the hippocampus, this brain region is a promising mediator linking differences in reproductive hormones to risk of depression. For instance, in the peripartum period, alongside an increased risk of depression diagnosis and robust hormonal changes (Pařízek et al., 2014; Schock et al., 2016; Stuebe et al., 2015), there are observable alterations to hippocampal volume in rodents, which return to normal in the weeks following parturition (Galea et al., 2000).

Previous studies have mainly focused on the correlation between reproductive hormones and whole hippocampal volume; testing the relationship at a single point in time (Barth et al., 2016; Panizzon et al., 2018). Although such studies are insightful, they are limited for three key reasons. Firstly, with correlations alone, it is not possible to discern whether reproductive hormones are causally affecting hippocampal volume, or whether reproductive hormones and hippocampal volume are impacted by an independent environmental factor (e.g. diet or stress). Secondly, as reproductive hormones are highly dynamic, it can be difficult, particularly in smaller studies with single measurements, to generate a stable quantitative measure that adequately captures inter-individual variation in hormone levels necessary to assess correlations with the hippocampus. Finally, until now, only whole hippocampal volumes have been assessed, but as certain areas of the hippocampus contain stem cells that give rise to new mature neural cells during AHN, it would be beneficial to evaluate individual subfield volumes in order to test whether the ‘neurogenic niches’ mediate volume differences in response to reproductive hormones, or whether it is mediated by other areas of the hippocampus.

To address these limitations, we employed a Mendelian randomization design, which uses genetic information to infer the presence of a causal relationship between two traits. We tested whether polygenic risk scores (PRSs) for reproductive hormones predict right or left hippocampal volumes; hippocampal subfield volumes; and subsequently MDD or PPD case-control status. This approach utilizes hormone-specific PRSs as stable genetic predictors of inter-individual variation in reproductive hormone levels. To achieve this, we used genome-wide association study (GWAS) summary statistics from 2906 individuals within the TwinsUK cohort, which has previously identified single nucleotide polymorphisms (SNPs) associated with circulating reproductive hormones, including estradiol, testosterone, progesterone and prolactin (Ruth et al., 2016). To identify links with hippocampal volume and depressive disorders we used the European RADIANT cohort consisting of 176 PPD cases, 2772 MDD cases and 1588 control participants, for which there is also a neuroimaging subset of 111 individuals. We identified the best combination of SNPs from a range of p-value thresholds obtained from each reproductive hormone GWAS, that predicted right or left hippocampal volume in our neuroimaging sample. Next, we tested the effect of this best-fit PRS on the 12 hippocampal subfield volumes. As these 111 individuals represented a subset of the larger depression case-control study, our final aim was to test whether this best-fit PRS for predicting hippocampal volumes, further predicted PPD or MDD in the full RADIANT cohort.

## 2 Materials and methods

### 2.1 RADIANT sample

RADIANT is an umbrella term for three studies which sought to understand genetic risk for MDD and factors aﬀ ;ecting response to antidepressant treatments (Lewis et al., 2010); this comprised of the Depression Network (DeNT) study (Farmer et al., 2004), the Depression Case-Control (DeCC) study (Cohen-Woods et al., 2009) and the Genome-Based Therapeutic Drugs for Depression (GENDEP) study (Uher et al., 2009). As the DeNT study was composed of sibling pairs, only one sibling from each family were randomly included in genetic studies (Lewis et al., 2010). The DeCC and DeNT studies only recruited individuals who had experienced recurrent depression. While the GENDEP study did not aim to solely recruit recurrent depression cases, 77.3% of cases were in fact recurrent. Therefore, the entire RADIANT cohort contains a majority of recurrent depression cases (94.6%). All participants were interviewed using the Schedules for Clinical Assessment in Neuropsychiatry (SCAN) interview (Wing et al., 1990) focusing on their two most severe depressive episodes (if applicable). Participants were excluded if they, or a first-degree relative, had ever experienced mania, hypomania, bipolar disorder, schizophrenia, schizo-affective disorder, intravenous drug dependence, substance-induced mood disorder or mood disorders secondary to medical illness or medication. A total of 2772 MDD cases, 176 PPD cases and 1588 unaffected controls were available from these studies for the work outlined here (Table 1).

**Table 1:** Demographic details of the neuroimaging subset and depressed patients and controls in the whole RADIANT cohort.

	*Neuroimaging subset*	*Entire RADIANT cohort*		
		*Control*	*PPD*	*MDD*
*N*	111	1588	176	2772
*Female (N, %)*	67 (60.4)	993 (62.5)	176 (100)	1901 (68.6)
*Age (mean, SD)*	50.0 (8.1)	42.4 (13.0)	41.6 (9.5)	46.9 (12.3)
*Depressed (N, %)*	58 (47.7)	–	–	–
*Antidepressants (N, %)*	44 (39.6)	–	–	–
*Handedness (N, %)*		–	–	–
Right	94 (84.7)	–	–	–
Left	13 (11.7)	–	–	–
Ambidextrous	3 (2.7)	–	–	–

Abbreviations: Antidepressants: Has taken antidepressant drugs within the past 6 months; PPD: postpartum depression; MDD: major depressive disorder.

Major depressive disorder cases: Participants were classified as MDD cases if they had experienced at least one episode of major depression of at least moderate severity as defined by the Diagnostic and Statistical Manual of Mental Disorders 4th edition operational criteria (DSM-IV) (American Psychiatric Association, 1994) or the International Classification of Diseases 10th edition operational criteria (ICD-10) (World Health Organization, 1992) for unipolar depression. For the purpose of this study we excluded MDD patients who met further criteria for PPD, as we considered these individuals separately. MDD cases were 68.6% female, with an age range 18–67 (mean = 46.9, S.D. = 12.3).

Postpartum depression cases: From the MDD cases identified within RADIANT (where data were available), cases of PPD were identified using the list of threatening experiences questionnaire (LTE-Q). The LTE-Q is a 12-term self-reported questionnaire which measures the occurrence of stressful life events in the 6 months prior to depression onset, to which childbirth has been added (Brugha and Cragg, 1990; Farmer et al., 2004). PPD cases were identified as females who had experienced either of their two most severe major depressive episodes within 6 months of childbirth. PPD cases were 100% female, with an age range of 20–67 (mean = 41.6, S.D. = 9.5).

Control participants: Control participants were screened using a modified version of the Past History Schedule (McGuffin et al., 1986) for any psychiatric disorder throughout their life. They were excluded if they, or a first-degree relative had suffered from depression or any other psychiatric disorder. Control participants were 62.5% female, with an age range of 18–89 (mean = 46.9, S.D. = 12.3).

Neuroimaging subset: For a subset of the participants described above (N = 111), neuroimaging data was available. Demographics of participants with both genotype and neuroimaging data are described in Table 1. This subset was 67% female with an age range of 26–66 years old (mean = 50.0, S.D. = 8.1). 58% of the neuroimaging cohort were depressed cases (either MDD or PPD) and all had experienced recurrent depression. 44% had taken pharmacological antidepressants in the 6 months preceding the MRI scan. The majority of the cohort were righthanded (94%). In addition to the exclusion criteria described above, participants were excluded from neuroimaging analysis if they had previously experienced any severe head trauma, neurological condition or any other contraindications to magnetic resonance scanning.

### 2.2 RADIANT genetic data

Genotyping data from the RADIANT cohort was already available and have been used in previous publications (Lewis et al., 2010). Genomic DNA was extracted from blood and buccal swabs as described previously (Freeman et al., 2003). DNA samples were genotyped using the Illumina Human610-Quad bead chip (Illumina, Inc., San Diego, CA, USA) at the Centre National de Genotypage (Evry Cedex, France). Single nucleotide polymorphism (SNP) information was available as PLINK files (Purcell et al., 2007).

### 2.3 RADIANT MRI data

MRI data had been collected from a subset of RADIANT, as described previously (Cole et al., 2011, 2013). Magnetization-Prepared Rapid Gradient Echo (MP-RAGE) T1-weighted scans were acquired using a 1.5 T General Electric Signa MR Imaging System (General Electric, Milwaukee, Wisconsin, USA) from participants in the sagittal plane using the parameters: echo time = 3.8 ms, repetition time = 8.592 ms, inversion time = 1000 ms, flip angle = 8°, voxel dimensions = 0.939 × 0.937 ×1.2 mm, matrix size 192 × 192, field of view = 240, slice thickness = 1.2 mm, number of slices = 180.

All T1-weighted images were visually inspected for motion artefact, wrap-around and grey/white contrast; no data were excluded. Automated whole brain segmentation, cortical reconstruction and hippocampal subfield segmentation were carried out using FreeSurfer v6.0 (Massachusetts General Hospital, Harvard Medical School; http://surfer.nmr.mgh.harvard.edu) (Fischl et al., 2002; Iglesias et al., 2015). Hippocampal subfield volumes were visually inspected and no manual edits were necessary. Volumes were also assessed so that outliers could be identified, although no data were excluded based on these measures. The hippocampal subfields identified using this segmentation method and included in this study are as follows: parasubiculum, presubiculum, subiculum, cornu ammonis 1 (CA1), cornu ammonis 2/3 (CA2/3), cornu ammonis 4 (CA4 or hilus), granule cell layer of the dentate gyrus, molecular layer of the dentate gyrus, fimbria, hippocampal-amygdala-transition area (HATA), hippocampal tail and hippocampal fissure. All subfields represent regions of grey matter except for the hippocampal fissure which is a sulcus residing between the dentate gyrus and subiculum.

### 2.4 Polygenic risk scoring

A polygenic risk score (PRS) is a number which represents an individual’s genetic load of risk alleles for a certain base trait. Here we created PRSs for estradiol, progesterone, prolactin and testosterone separately for each individual in the RADIANT cohort. PRSs for each reproductive hormone were generated using summary statistics from the largest GWAS for plasma reproductive hormones to-date, consisting of 2906 individuals from the Twins UK cohort (Ruth et al., 2016). This GWAS used a sample consisting of predominantly female participants, and excluded individuals undergoing any hormonal therapies. The original GWAS analysis took into account the age, sex, BMI, stage of menstrual cycle and menopausal status of the participants.

PRSice version 1.25 software was used to implement a pipeline of processes common in PRS creation (Euesden et al., 2015). Firstly, SNPs present in only the base GWAS or target RADIANT cohort were removed. Ambiguous (A/T or C/G) SNPs were also removed. SNPs in linkage disequilibrium (r^2^>0.1) were removed using a process called clumping, leaving a single SNP in each 250 kb LD window with the smallest p-value from the GWAS. Clumping was preferred over pruning in order to retain SNPs across the entire genome. The remaining SNPs were used to calculate PRSs for eight p-value thresholds (0.001, 0.01, 0.05, 0.1, 0.2, 0.3, 0.4 and 0.5). At each p-value threshold, the SNPs that fell below this threshold in the GWAS summary statistics were identified, and the number of risk variants (0, 1 or 2) that an individual carried was multiplied by the logarithm of the odds ratio for that variant. The sum of all these values gives the PRS for an individual and the process is repeated for all the individuals in the cohort and for all hormones.

### 2.5 Statistical analyses

#### 2.5.1 PRSs and hippocampal volumes

Left and right whole hippocampus and hippocampal subfield volumes were checked for normality using histograms and Kolmogorov-Smirnov and Shapiro-Wilk normality tests (Vetter, 2017), with non-normal volumes undergoing log-transformation. Subsequently, volumes were adjusted for intracranial volume, sex, age and depression case-control status by taking the standardized residuals (z-scores) using SPSS v.24. The effects of handedness in the whole cohort, and current antidepressant use (last 6 months) amongst depressed cases, was tested, and found not to significantly affect either right or left hippocampal volumes (p>0.05).

We first tested the relationships between PRSs for each reproductive hormone, and either adjusted left or right hippocampal volume, using regressions in PRSice (Euesden et al., 2015). In each regression we included seven population covariates to correct for population structure. These population covariates were the top seven components derived using multi-dimensional scaling in PRSice. We tested the predictive power of PRSs derived from SNPs under eight p-value thresholds (p = 0.001, 0.01, 0.05, 0.1, 0.2, 0.3, 0.4 and 0.5), in order to determine the optimal p-value threshold and “best fit” PRS for each hormone and hemisphere combination. For each hormone and hemisphere combination, we corrected for the number of p-value thresholds tested using the Benjamini-Hochberg correction (Benjamini and Hochberg, 1995) and a false discovery rate of 10%.

Where a PRS for a reproductive hormone explained a significant proportion of the variance in either whole left/right hippocampal volume, we tested whether the same best fit PRS predicted the volume of its 12 constituent subfields. This was again performed using PRSice software, with the same seven population covariates, as described above. We corrected for the number of subfields tested using the Benjamini-Hochberg correction with a false discovery rate of 10%.

#### 2.5.2 PRS and depression case-control status

Lastly, once we had identified a hormone-related PRS which significantly predicted hippocampal volume, we tested whether the same PRS was associated with PPD or MDD case-control status in the wider RADIANT cohort. Binary logistic regressions, including sex-stratified regressions were performed for each depression subtype in PRSice, covarying for seven population covariates, derived using multi-dimensional scaling in PRSice, with sex included as a covariate where appropriate (i.e. when both males and females were included in the same analysis).

## 3 Results

### 3.1 Polygenic risk for circulating estradiol is associated with whole hippocampal volume

**Fig. 1:**
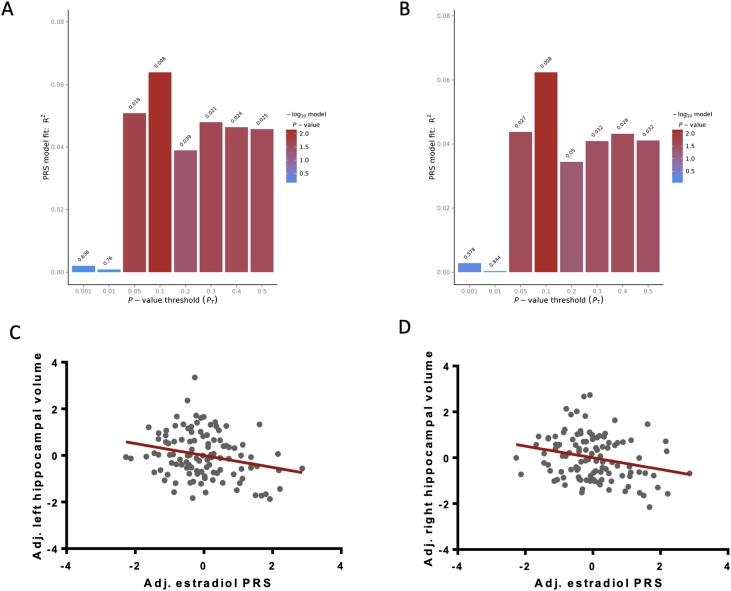
A&B) Output from PRSice displaying the results of regressing the estradiol PRS against the (A) left and (B) right hippocampal volume at the range of p-value thresholds tested (x-axis). A combination of SNP information under p=0.1 significantly best predicted both left and right hippocampal volumes. Variance explained (R^2^) is indicated on the y-axis and uncorrected p-values are indicated above each bar. C&D) Scatterplots demonstrating significant negative correlations between the best-fit PRS for estradiol levels (z-scores; adjusted for 7 population covariates derived from multidimensional scaling to control for population stratification) and whole hippocampal volumes (z-scores; adjusted for intracranial volume, age, sex, case-control status) for the (C) left and (D) right hemispheres. (For interpretation of the references to colour in this figure legend, the reader is referred to the web version of this article).

We created PRSs for plasma estradiol, testosterone, progesterone and prolactin at eight p-value thresholds and tested for their association with either right or left whole hippocampal volume. After correcting for multiple p-value thresholds, only the PRS for estradiol was found to be significantly associated with whole hippocampal volume (Table 2). The best-fit estradiol PRS (using p_thresh_ = 0.1 for both hemispheres) was negatively associated with both right (B = −2498.209, SE = 919.795, p = 0.008, R^2^ = 0.062) and left (B = −2528.269, SE = 928.911, p = 0.008, R^2^ = 0.064) hippocampal volumes, and survived our multiple testing criteria (q < 0.1) (Table 2, Fig. 1). The best fit PRSs for prolactin, testosterone and progesterone were not significantly associated with either right or left whole hippocampal volume.

**Table 2:** Table showing the results of regressions that tested the predictive ability of best-fit PRS for all reproductive hormones on left and right whole hippocampal volumes.

Hippocampus	Optimal p-value threshold	nSNPs	p-value	q-value	R^2^	β	SE
Estradiol							
Right	0.1	27389	0.008	*0.051	0.062	−2498.209	919.795
Left	0.1	27389	0.008	*0.040	0.064	−2528.269	928.911
Progesterone							
Right	0.001	275	0.120	0.286	0.022	−112.957	72.091
Left	0.01	2340	0.072	0.253	0.030	−370.014	203.569
Prolactin							
Right	0.01	2520	0.339	0.735	0.008	370.970	386.164
Left	0.4	54198	0.055	0.176	0.034	5977.305	3083.026
Testosterone							
Right	0.05	9953	0.096	0.543	0.025	−1274.616	758.934
Left	0.05	9953	0.138	0.586	0.020	−1148.102	768.731

The optimal threshold is defined as the p-value threshold for the group of SNPs which produces the best-fit PRS for each regression. Whole hippocampal volume was corrected for intracranial volume, age, sex and depression status. All regression analyses include seven population covariates, derived from multidimensional scaling, to control for population stratification. Associations which survive FDR corrections are indicated with *. Abbreviations: PRS: polygenic risk score; nSNPs: number of SNPs included in the optimal PRS; p-value: uncorrected p-value; q-value: FDR-corrected p-value; R^2^: amount of variance explained by the respective optimal PRS; *β*: regression coefficient; SE: standard error.

### 3.2 Polygenic risk for circulating estradiol is associated with hippocampal subfields

**Fig. 2:**
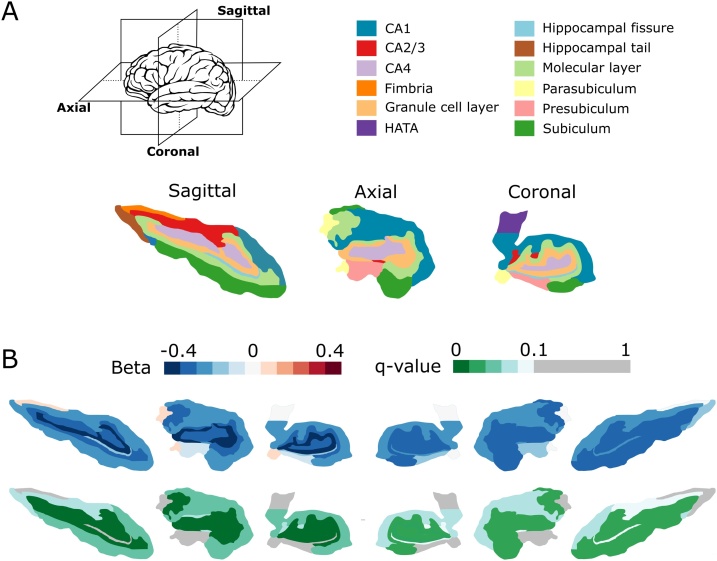
Visual representation of the predictive ability of the best-fit PRS for estradiol on hippocampal subfield volume. A. Example sagittal, axial and coronal cross-sections of the human hippocampus segmented from an example MRI image using FreeSurfer and colour-coded to indicate each hippocampal subfield. B. For each hemisphere and subfield combination, the results of the linear regression are represented in a scale of dark blue (β=−0.4), through white (β=0), to dark red (β=0.4) for the beta coefficient, and green to white for the FDR-corrected q-value. Relationships which did not survive the FDR correction are represented in grey (q>0.1). The six figures to the left relate to the left hippocampus, and the six figures to the right relate to the right hippocampus. Relative hippocampal subfield structure is for visualisation only and is not representative of all participants. The numerical data is available in Table 3.

Next, we investigated the association between the best-fit estradiol PRS and hippocampal subfield volumes. As with the whole hippocampus, the estradiol PRS was associated with the volume of multiple hippocampal subfields (Table 3). After multiple testing correction, the estradiol PRS was significantly associated with the right and left subiculum, CA1, molecular layer of the dentate gyrus, granule cell layer of the dentate gyrus, CA2/3 and CA4 regions (Table 3). It was also unilaterally associated with the volume of the left hippocampal tail and right hippocampal fissure. A graphical representation of the associations between the estradiol PRS and all hippocampal subfield volumes can be observed in Fig. 2. For all significant relationships, a PRS indicative of higher estradiol levels was associated with a reduced volume.

**Table 3:** Table showing the results of regressions that test the predictive ability of best-fit PRS for estradiol, on hippocampal subfield volumes.

Subfield	Uncorrected p-value	q-value	R^2^	β	SE
Right hippocampus					
CA1	0.026	*0.063	0.043	−2120.714	941.310
CA2/CA3	0.051	*0.087	0.035	−1870.666	945.199
CA4	0.009	*0.034	0.060	−2451.741	919.490
Granule cell layer	0.010	*0.034	0.058	−2398.323	916.184
Molecular layer	0.004	*0.034	0.068	−2690.309	912.037
Subiculum	0.011	*0.034	0.053	−2394.581	928.655
Presubiculum	0.147	0.196	0.016	−1399.802	958.286
Parasubiculum	0.784	0.940	0.001	−268.661	975.640
Hippocampal tail	0.085	0.127	0.028	−1657.922	952.250
Hippocampal fissure	0.043	*0.086	0.027	−1952.554	952.076
Fimbria	0.996	0.996	0.000	−4.561	945.580
HATA	0.962	0.996	0.000	45.173	941.158
Left hippocampus					
CA1	0.025	*0.060	0.045	−2129.952	935.255
CA2/CA3	0.038	*0.075	0.040	−1995.406	950.145
CA4	0.002	*0.013	0.082	−2860.001	914.747
Granule cell layer	0.001	*0.011	0.094	−3062.294	898.117
Molecular layer	0.003	*0.013	0.077	−2774.238	922.557
Subiculum	0.016	*0.049	0.052	−2284.700	934.863
Presubiculum	0.416	0.554	0.006	−787.328	963.220
Parasubiculum	0.473	0.568	0.005	679.613	944.346
Hippocampal tail	0.044	*0.075	0.038	−1935.976	946.996
Hippocampal fissure	0.105	0.158	0.025	−1581.2	968.023
Fimbria	0.525	0.573	0.004	619.043	971.228
HATA	0.994	0.994	0.000	−7.131	967.600

Each hippocampal volume was adjusted for intracranial volume, age, sex and depression status. All regression analyses include seven population covariates, derived from multidimensional scaling, to control for population stratification. Associations which survive FDR corrections are indicated with *. Abbreviations: p-value: uncorrected p-value; q-value: FDR-corrected p-value; R^2^: amount of variance explained by the respective optimal PRS; *β*: regression coefficient; SE: standard error.

### 3.3 Polygenic risk for circulating estradiol does not predict depression case-control status

Finally, we tested whether the best-fit PRS for estradiol levels, identified above, predicted the risk of depressive disorders. The estradiol PRS was not associated with PPD case-control status or MDD case-control status, including in sex-stratified analyses (p>0.05; Table 4).

**Table 4:** Table showing the results of regressions that test the predictive ability of best-fit PRS for estradiol and hippocampal volume on case control status for either postpartum depression of major depressive disorder in males and females. All regression analyses include seven population covariates, derived from multidimensional scaling, to control for population stratification.

*Depressive disorder*	*P*	*Adj. R^2^*	*β*	*SE*
**PPD vs all controls**	0.121	0.00282	−1211.691	782.234
PPD vs female controls	0.179	0.00266	−1076.882	802.015
**MDD vs all controls**	0.595	0.00008	169.200	318.049
Female MDD vs female controls	0.624	0.00005	193.213	393.920
Male MDD vs male controls	0.807	0.00011	132.561	542.234

Abbreviations: PRS: polygenic risk score; Adj. R^2^: amount of variance explained by the respective optimal; *β*: regression coefficient; SE: standard error; PPD: postpartum depression; MDD: major depressive disorder.

## 4 Discussion

This study sought to better understand the relationship between circulating reproductive hormones and hippocampal volume via the application of polygenic epidemiology, whereby we estimated inter-individual variation in hormone levels based on genetic data, and tested how this affected hippocampal subfield volumes. We further investigated whether the same polygenic signature was associated with depressive disorders. Our results demonstrate a negative association between polygenic risk for estradiol levels and whole hippocampal volume, as well as the volume of many of its constituent subfields. However, this polygenic risk score did not predict the occurrence of depressive disorders. Previous literature has linked estradiol levels to whole hippocampal volume in humans, but this is the first to infer causality via polygenic scoring.

The hippocampus is a major target of estrogens in the brain due its high density of estrogen receptors which have the capacity to influence hippocampal volume through multiple actions, including altered gene expression, differential methylation of estrogen-responsive genes or by rapid-non genomic signaling pathways (Duarte-Guterman et al., 2015; Guintivano et al., 2014; Sárvári et al., 2015; Soma et al., 2018). Our results reveal an enrichment of estradiol’s effect on ‘neurogenic regions’ of the hippocampus, namely the granule cell layer and molecular layer of the dentate gyrus. This suggests that polygenic risk for estradiol levels may confer some of its long-term influences on hippocampal volumes via moderating AHN. Changes in AHN have been associated with volumetric changes in the CA1 in rodents due to altered dendritic branching (Schoenfeld et al., 2017), which could feasibly also impact the CA4 region due to the presence of mossy fibers from dentate gyrus granule cells (Scharfman and Myers, 2013). In general, rodent studies indicate a proliferative effect of estradiol on dentate gyrus neural stem cells, but the majority of these studies focus on the very short-term effects of large changes in estradiol (Mahmoud et al., 2016). Therefore, it is unclear what impact small, long-term differences in estradiol may have on the neurogenic potential of the hippocampus.

In the context of previous research, our findings support reports which have revealed an association between higher estradiol levels and smaller hippocampal volumes (Heijer et al., 2003; Seiger et al., 2016). It also agrees with the observation of reduced hippocampal volume during pregnancy in rats, when estradiol, alongside other sex hormones, is elevated (Galea et al., 2000; Rolls et al., 2008). However, they are in contrast to other studies which have shown that higher estradiol concentration is associated with larger hippocampal volumes and propose a neuroprotective effect of estradiol (Barth et al., 2016; Bayer et al., 2013; Galea et al., 2013; Kesler et al., 2004). This discrepancy could be due to a number of different reasons. For example, the dynamic and acute effects of estradiol, which are more commonly studied, may be different to the more chronic, stable, and possibly lifetime effects captured by polygenic risk scores used in this study. Indeed, many of these studies quantified hippocampal volume in the post-menopausal period, following estradiol supplementation or alongside the menstrual cycle. Alternatively, as many previous studies have employed *in vitro* or animal model systems to draw conclusions about estradiol’s effects, it may be the case that estradiol has different effects *in vivo* in humans. Finally, the majority of human studies conducted have focused on the impact of estradiol in either elderly, disease or medicated states. Therefore, it is difficult to discern what impact naturally occurring long-term differences in endogenous estradiol levels could have on the hippocampus.

Future research will be needed to better understand the differences in estradiol’s acute and chronic actions in a variety of ages. Different stages of the lifespan are characterized by large changes in estradiol levels, including puberty, pregnancy and the menopause. Therefore, future work should aim to determine how applicable these findings are to these different contexts. Finally, this result does not necessarily reject the potential neuroprotective effects of estradiol in the menopause or in neurodegenerative diseases. In these cases, exogenous estradiol is used to rectify large deficits in endogenous estradiol which would not be present in the study population used here (Raz et al., 2004). Additionally, many of the benefits of exogenous estradiol in neurogenerative diseases are independent of hippocampal volume changes (Arevalo et al., 2015). Without fully understanding the cellular mechanism linking estradiol PRS to hippocampal volume observed in this study, we cannot say whether our findings are relevant to these therapeutic interventions.

We do not observe an association between PRSs for testosterone, progesterone or prolactin and hippocampal volume in our study. Compared to estradiol, the evidence for an association between hippocampal volume and these other reproductive hormones is far weaker. While there is some evidence that plasma testosterone correlates with hippocampal volume, this has only been conducted in males and tends to be in disease, elderly or medicated states (Foland-Ross et al., 2019; Panizzon et al., 2010; Wainwright et al., 2011). There are very few human studies investigating the role of progesterone or other progestins in hippocampal volume and many studies are confounded by concurrent changes in estradiol levels (Pletzer et al., 2018). Furthermore, it appears that endogenous progesterone compared to synthetic progestins have very different impacts on the brain (Chan et al., 2014). Like progesterone, the link between plasma prolactin and the hippocampus has not been properly investigated in humans. While there is a large body of work suggesting that prolactin can impact the rodent hippocampus at the cellular level (Cabrera-Reyes et al., 2017; Carretero et al., 2018; Morales et al., 2014), this does not appear to translate to any change in whole hippocampal volume (Torner et al., 2009). There is evidence from animal and cellular models that reproductive hormones can influence the hippocampus at the cellular level through changes in AHN, cell survival or branching (Chan et al., 2014; Morales et al., 2014; Ransome and Boon, 2015), therefore it is possible that this may also occur in humans without affecting whole hippocampal volume.

In the context of MDD and PPD, PRS for estradiol did not directly relate to case-control status. This suggests that although estradiol could be one factor accounting for hippocampal volume reductions commonly observed in psychiatric patients, it does not directly predict case-control status. Consequently, it may be interesting to test if the estradiol PRS predicts other diseases in which the hippocampus has been linked, for example schizophrenia (Riecher-Rössler, 2017). This idea is supported by the observed link between estradiol PRS and hippocampal subfields such as the CA regions, subiculum and dentate gyrus in our study, which are commonly atrophied in a variety of psychiatric and degenerative disorders (Arnold et al., 2015; Hanseeuw et al., 2011; Haukvik et al., 2015; Papiol et al., 2017). Our negative result suggests that baseline estradiol levels may not play a causal role in depression, but this is not to say that estradiol is not involved in the development of depressive disorders. We acknowledge that inter-individual variation explaining estradiol levels in our base cohort may be different from those during pregnancy or other life events when plasma estradiol changes significantly. Additionally, environmental factors such as breastfeeding, parity or the drop following parturition may be more pertinent in moderating reproductive hormones in the peripartum period than genetic contributors (Bonnar et al., 1975; Schock et al., 2016; Zhang et al., 2016). Our approach also utilized a stable, genetic predictor of hormone levels and therefore ignores any hormonal fluctuations that occur throughout the lifespan and are thought to contribute to depressive states (Gordon et al., 2016). Furthermore, inter-individual variation in response to altered hormone levels, which are not considered here, have been shown to contribute to depressive disorders (Bloch et al., 2000).

Despite the important findings detailed here, our study has three key limitations. Firstly, the accuracy of the PRS is subject to the power of both the original GWAS and our target datasets. Although the original GWAS was the largest to-date, it is likely still underpowered, and consequently our PRS may lack predictive power. Furthermore, the neuroimaging dataset and PPD subsets are small, which may mean we are unable to detect smaller effect sizes in our sample. In addition, our neuroimaging cohort consisted of both healthy and depressed individuals. Although we detected no differences in volume between those who had and had not been on antidepressants within the last six months, we were underpowered to test the effects of individual drug types and treatment durations, which have been linked to changes in hippocampal volume (Boldrini et al., 2013). Additionally, we lacked information on factors such as menstrual stage, menopause or hormonal medications, which may also impact hippocampal volume (Barth et al., 2016; Bayer et al., 2013). Secondly, although PRSs are commonly used in Mendelian randomization designs and genetic epidemiology (Dudbridge, 2013; Papiol et al., 2017; Peyrot et al., 2014), they may be subject to the effects of horizontal pleiotropy; whereby a subset of SNPs included in the PRS predict variance for another related trait, driving the observed association (Dudbridge, 2013). Although horizontal pleiotropy is possible, it’s important to note that at the protein level, estradiol is correlated with testosterone (Ruth et al., 2016), which did not predict hippocampal volume in our study; supporting the view that the effect may be uniquely related to estradiol. In the future, inference of causality can be made more certain once more powerful GWASs are performed and replicable genome-wide significant association hits can be used as an instrumental variable to probe the direction of association between estradiol and hippocampal volume. Finally, the definition of PPD within this study was made retrospectively using a subset of the MDD cohort. We therefore may be missing other PPD cases due to insufficient information (e.g. where pregnancy aligned to the third most severe depressive episode, which was not captured). Additionally, for our PPD analysis we used a control population which did not exclude women who had not experienced a pregnancy and therefore could not have experienced PPD. We did this primarily due to a small sample size and missing data in our control sample on whether females had experienced a previous pregnancy. Subsequently, because the risk for PPD is relatively low on the population level, we included all female RADIANT controls in order to achieve the best power possible. We acknowledge that screened controls would have made for a more ideal comparison group.

## 5 Conclusions

Our study employed novel genetic and neuroimaging analyses which provide new insight into the regulation of hippocampal volume by estradiol. Our work suggests genetic risk for higher estradiol levels predict smaller hippocampal volumes, possibly mediated via changes to AHN in the neurogenic regions of the hippocampus. Genetic risk for higher estradiol levels was not, however, associated with risk for developing MDD or PPD. Future studies should aim to replicate our findings in larger samples, test the generalizability of the findings outside of the European population, and examine whether estradiol’s effects on the hippocampus moderate risk for diseases other than depression.

## Funding

Demelza Smeeth’s PhD studentship is sponsored by the Guy’s and St Thomas’ Charity. Timothy R. Powell is funded by a Medical Research Council (MRC) Skills Development Fellowship (MR/N014863/1). Rudolf Uher is supported by the Canada Research Chairs Program (file number 950-225925) and the Canadian Institutes of Health Research (funding reference numbers 124976, 142738 and 148394). This work was funded by a joint grant from the MRC, UK and GlaxoSmithKline [G0701420], and by the National Institute for Health Research (NIHR) Mental Health Biomedical Research Centre at South London and Maudsley NHS Foundation Trust and King’s College London. GlaxoSmithKline funded the collection of the DeNt cohort of depression cases, and the genotyping of all RADIANT cases (with the MRC). The GENDEP study was funded by a European Commission Framework 6 grant, EC Contract Ref.: LSHB-CT-2003-503428. The views expressed are those of the authors and not necessarily those of the NHS, the NIHR or the Department of Health and Social Care. The funding sources had no role in the study design, in the collection, analysis, and interpretation of data, or in the writing of the report and the decision to submit the article for publication.
